# Human papillomavirus genotype distribution in Madrid and correlation with cytological data

**DOI:** 10.1186/1471-2334-11-316

**Published:** 2011-11-15

**Authors:** Paloma Martín, Linah Kilany, Diego García, Ana M López-García, Mª José Martín-Azaña, Victor Abraira, Carmen Bellas

**Affiliations:** 1 Molecular Pathology Laboratory, Department of Pathology, Hospital Universitario Puerta de Hierro - Majadahonda, Madrid, Spain; 2 Cytopathology Laboratory, Department of Pathology, Hospital Universitario Puerta de Hierro - Majadahonda, Madrid, Spain; 3 Department of Gynaecology, Hospital Universitario Puerta de Hierro - Majadahonda, Madrid, Spain; 4 Unidad de Bioestadística Clínica, Hospital Universitario Ramón y Cajal, IRYCIS, CIBER Epidemiología y Salud Pública (CIBERESP), Madrid, Spain

## Abstract

**Background:**

Cervical cancer is the second most common cancer in women worldwide. Infection with certain human papillomavirus (HPV) genotypes is the most important risk factor associated with cervical cancer. This study analysed the distribution of type-specific HPV infection among women with normal and abnormal cytology, to assess the potential benefit of prophylaxis with anti-HPV vaccines.

**Methods:**

Cervical samples of 2,461 women (median age 34 years; range 15-75) from the centre of Spain were tested for HPV DNA. These included 1,656 samples with normal cytology (NC), 336 with atypical squamous cells of undetermined significance (ASCUS), 387 low-grade squamous intraepithelial lesions (LSILs), and 82 high-grade squamous intraepithelial lesions (HSILs). HPV detection and genotyping were performed by PCR using 5'-biotinylated MY09/11 consensus primers, and reverse dot blot hybridisation.

**Results:**

HPV infection was detected in 1,062 women (43.2%). Out of these, 334 (31%) samples had normal cytology and 728 (69%) showed some cytological abnormality: 284 (27%) ASCUS, 365 (34%) LSILs, and 79 (8%) HSILs. The most common genotype found was HPV 16 (28%) with the following distribution: 21% in NC samples, 31% in ASCUS, 26% in LSILs, and 51% in HSILs. HPV 53 was the second most frequent (16%): 16% in NC, 16% in ASCUS, 19% in LSILs, and 5% in HSILs. The third genotype was HPV 31 (12%): 10% in NC, 11% in ASCUS, 14% in LSILs, and 11% in HSILs. Co-infections were found in 366 samples (34%). In 25%, 36%, 45% and 20% of samples with NC, ASCUS, LSIL and HSIL, respectively, more than one genotype was found.

**Conclusions:**

HPV 16 was the most frequent genotype in our area, followed by HPV 53 and 31, with a low prevalence of HPV 18 even in HSILs. The frequency of genotypes 16, 52 and 58 increased significantly from ASCUS to HSILs. Although a vaccine against HPV 16 and 18 could theoretically prevent approximately 50% of HSILs, genotypes not covered by the vaccine are frequent in our population. Knowledge of the epidemiological distribution is necessary to predict the effect of vaccines on incidence of infection and evaluate cross-protection from current vaccines against infection with other types.

## Background

The association of human papillomavirus (HPV) infection with cervical cancer and its precursor lesions is well established [1,2]. More than one hundred HPV genotypes have been described, about 40 of which can infect mucosal epithelia [3], causing mainly transient infections [4].

Based on the etiological role in the progression to cervical cancer, 15 HPV genotypes have demonstrated strong oncogenic potential and are classified as high-risk HPVs [5]. Persistent infection with oncogenic types of HPV is considered the main risk factor associated with this disease. The HPV genotypes and prevalence found in different regions of the world vary both in type and incidence, probably as a consequence of the many factors associated with HPV infection [6]. In a substantial percentage of HPV infections, two or more genotypes may be found. About 20-50% of the infected women carry more than one viral type [7,8]. For some groups, co-infection with multiple HPV types correlates with the severity of cervical intraepithelial neoplasia [9]. In contrast, other authors [10,11] have reported that infection with multiple types does not increase the risk of developing or progression of cervical neoplasia. HPV typing has important prognostic and therapeutic value and the recent development of HPV vaccines makes it increasingly more important. It has been suggested that the impact of the vaccines could vary depending on the regional distribution of HPV types and co-infection patterns. To investigate the potential impact of vaccination strategy, it is essential to assess the local distribution of HPV types and the incidence of co-infections. The aim of the present study was to investigate the distribution of HPV subtypes and its relationship with cervical lesions in women in Madrid, Spain.

## Methods

### Specimen preparation

A total of 2,461 cytological specimens from May 2006 to June 2010 were included in this study. Patients were selected if both molecular analysis and cytology were available and if no previous HPV study had been performed. Cytological samples corresponded to patients attending the specialised gynaecology outpatient clinics of the Hospital Universitario Puerta de Hierro, Madrid, Spain. These patients were selected on the basis of a previous abnormal Pap smear. A second sample was obtained, for which, exfoliated cervical cells were obtained using a cytobrush and collected in PreservCyt Solution (Cytyc Corporation, Malborough, MA). The slides were prepared with a TP2000 (Cytyc) instrument as recommended by the manufacturer. ThinPrep cytological slides were screened and the adequacy and the degree of abnormality were assessed using the criteria set out in The Bethesda System 2001 guidelines http://www.asccp.org/ConsensusGuidelines/2001BethesdaSystem/tabid/5969/Default.aspx. Residual liquid material from ThinPrep samples was used for HPV detection. One millilitre of each collected PreservCyt sample was aliquoted with an individual sterile pipette for molecular study. DNA was extracted with QIacube DNA Investigator Kit (Qiagen, Hilden, Germany) in accordance with the manufacturer's instructions.

### HPV genotyping

HPV DNA detection and genotyping were performed by PCR using the L1 consensus HPV PGMY09/PGMY11 primer set [12], and flow-through hybridisation (HybriBio Limited, Hong Kong) as described previously [13,14]. It generates an amplicon that is approximately 450 bp long and appropriate for type identification. Positive and negative controls were used in each PCR. The gene chip contained type-specific oligonucleotides immobilised on a nylon membrane. The final results were detected by colorimetric changes on the chip under direct visualisation. HPV types included in the kit were: high-risk genotypes: 16, 18, 31, 33, 35, 39, 45, 51, 52, 56, 58, 59, 66, and 68; low-risk: 6, 11, 42, 43 and 44; and probable high-risk: 53 and 81.

### Statistical analysis

HPV genotype distribution analysis was performed using SPSS version 15.0. The χ^2 ^test was used to evaluate statistical significance between genotype, cytological lesion and age. The median age of 33 years was chosen to dichotomise age in the analysis of multiple genotypes infection. The study protocol was approved by our institutional review board and informed consent was obtained in each case.

## Results

There were 2,461 samples from women aged 15-75 years analysed for HPV infection from May 2006 to June 2010: 1,399 (56.8%) were negative for HPV and 1,062 (43.2%) were positive. The cytological diagnosis was: 1,656 (67.3%) with normal cytology (NC); 336 (13.7%) with atypical squamous cell of undetermined significance (ASCUS); 387 (15.7%) low-grade squamous intraepithelial lesions (LSILs); and 82 (3.3%) high-grade squamous intraepithelial lesions (HSILs). The percentage of cases positive for HPV infection in the different diagnostic categories was: 20% in NC, 84% in ASCUS, 94% in LSILs, and 96% in HSILs (Table 1).

**Table 1:** Association between cytological lesions and HPV positivity

Lesion	HPV negative, n (%)	HPV positive, n (%)
NC (n = 1656)	1322 (79.8%)	334 (20.2%)
ASCUS (n = 336)	52 (15.5%)	284 (84.5%)
LSIL (n = 387)	22 (5.7%)	365 (94.3%)
HSIL (n = 82)	3 (3.7%)	79 (96.3%)

The current analysis focused on the 1,062 HPV-positive samples to establish the distribution of HPV subtypes in our area. Cytological diagnosis of these HPV-positive samples included 334 (31%) from women with negative cytology and 728 (69%) with some cytological abnormality. The cytological diagnosis of abnormal cases was as follows: 284 cases (27%) with ASCUS, 365 (34%) with LSILs, and 79 (8%) with HSILs.

Mean age of the patients was 34 ± 10.5 years (range 15-75). Median age of NC patients was 34.5 years, 34.2 years for ASCUS, 32.3 years for LSILs, and 39.6 years for HSILs. The distribution of the cytological diagnoses with regard to age is shown in Table 2. Women older than 32 years had the following cytological distribution: 33% NC, 25% ASCUS, 30% LSIL, and 12% HSIL, whereas in women younger than 33 years, only 4% had HSILs, 39% LSILs, 28% ASCUS and 29% NC.

**Table 2:** Distribution of cytological diagnosis in HPV-positive samples with regard to age

Lesion	≥33 yrs n = 499	< 33 yrs n = 528
NC	33.3%	28.8%
ASCUS	25.5%	27.8%
LSIL	29.7%	39.6%
HSIL	11.5%	4.0%

χ^2 ^= 30.22; p = 0.000

The most common HPV types in the complete series were as follows: 16 (28%), 53 (16%), 31 (12%), 52 (10%), 6 (9%), 18 (8%), 58 (8%), and 66 (8%). The prevalence of other HPV types was: 33 (5%), 39 (5%), 56 (5%), 81 (5%), 42 (4%), 45 (4%), 11 (3%), and 51 (3%). The most common HPV types in cytologically normal women were: 16 (21%), 53 (16%), 6 (10%), 31 (10%) and 52 (8%). HPV18 was found in 6% of NC cases. In abnormal samples, the distribution was as follows (Table 3). ASCUS: 16 (31%), 53 (16%), 31 (11%), 52 (11%) and 6 (10%); LSILs: 16 (26%), 53 (19%), 31 (14%), 18 (11.5%), 52 (11%) and 66 (11%); and HSILs: 16 (51%), 52 (14%), 31 (11%), 33 (10%) and 58 (8%). The three most frequent HPV types were 16, 53 and 31 in NC, ASCUS and LSILs, respectively.

**Table 3:** Distribution of HPV genotypes found in each cytological diagnosis

	6	11	16	18	31	33	39	42	45	51	52	53	56	58	66	81	Others
**NC (n = 334)**	10.2% (34)	2.1% (7)	21% (70)	6.3% (21)	10.5% (35)	4.8% (16)	5.4% (18)	4.2% (14)	3.6% (12)	1.2% (4)	8.4% (28)	16.2% (54)	2.4% (8)	7.5% (25)	6% (20)	4.8% (16)	15% (50)
**ASCUS (n = 284)**	9.9% (28)	3.9% (11)	30.6% (87)	7.7% (22)	11.3% (32)	4.2% (12)	5.3% (15)	3.2% (9)	4.9% (14)	1.1% (3)	10.9% (31)	15.8% (45)	3.2% (9)	7% (20)	7% (20)	6.3% (18)	13% (37)
**LSIL (n = 365)**	10.1% (37)	3.8% (14)	26.3% (96)	11.5% (42)	14.2% (52)	3.8% (14)	5.2% (19)	7.1% (26)	3.3% (12)	8.5% (31)	10.7% (39)	18.9% (69)	7.9% (29)	10.1% (37)	11.5% (42)	5.2% (19)	16.2% (59)
**HSIL (n = 79)**	1.3% (1)	0	50.6% (40)	5.1% (4)	11.4% (9)	10.1% (8)	1.3% (1)	0	2.5% (2)	1.3% (1)	13.9% (11)	5.1% (4)	5.1% (4)	7.6% (6)	1.3% (1)	3.8% (3)	6.3% (5)

Single infection (one HPV type) was observed in 696 (65.5%) samples, and 366 (34.5%) patients were infected with multiple HPV types. Double infections (two different genotypes) accounted for 60% of the multiple infections, three genotypes were detected in 26% of these 366 samples, and four or more types in 14%. In women under 33 years old, infections due to more than one HPV type were found in 39.6% of cases, whereas in those older than this, the prevalence was 26.7% (p < 0.01). HPV 16 was the most common genotype detected in co-infections, and was present in 38% of cases, followed by HPV 53 (27%) and HPV 31 (20%). Co-infections were found in 25% of NC, 36% of ASCUS, 45% of LSIL, and 20% of HSIL cases (Table 4). Distribution of cytological lesions in those 366 samples was as follows: 23% NC, 28% ASCUS, 45% LSIL, and 4% HSIL. Single infection in HSIL samples was found in 80% of cases (63 samples), and HPV 16 was detected in 50% of them. Other genotypes found in HSIL single infection were HPV 31 (9.5%), HPV 33 (7.9%), HPV 52 (6.3%), and HPV 58 (4.8%). HPV 18 as single infection in HSILs was found in one case and in three cases as co-infection with HPV 52.

**Table 4:** Correlation between HPV co-infection and cytological lesions

Cytological lesion	Mono-infection	Co-infection
NC (n = 334)	251 (75.1%)	83 (24.9%)
ASCUS (n = 284)	183 (64.4%)	101 (35.6%)
LSIL (n = 365)	199 (54, 5%)	166 (45.5%)
HSIL (n = 79)	63 (79.7%)	16 (20.3%)
**Total**	**696 (65.5%)**	**366 (34.5%)**

χ^2 ^= 40.49; p = 0.000

The global results demonstrated that HPV 16 was the most prevalent infection (28%), whereas HPV 18 was found more rarely (8%).

## Discussion

Our specimens came from a group of women who attended a cervical pathology unit because of a previous abnormal Pap test result. We selected the HPV-positive cases to investigate the genotype distribution in our area. Several studies have been published concerning genotype distribution in several Spanish regions [14-18], but few data are available about the distribution in Madrid [19]. The prevalence and distribution of HPV genotypes varies greatly worldwide, and these differences might be related to the complex geographical and biological interplay between different HPV types and host immunogenetic factors [20].

Consistent with previously published data, genotype HPV 16 was the most common type in our series, and was detected in 28% of samples. With the exception of Eastern Africa, China, Japan and Taiwan [21-23], HPV 16 is the most prevalent type in all parts of the world. Our study showed that HPV 53 was the second most common genotype (16% in the whole series), which is similar to previous Italian [24,25] and Spanish [17,19,26] studies, however, HPV 53 is not a frequent genotype in other Mediterranean countries, such as Greece and Turkey [27,28]. The epidemiological patterns of HPV type prevalence could differ depending on the region of origin. Another factor that could influence the genotype distribution is the primer system used. The GP5+/6+ primer set is less sensitive for HPV 53 detection, whereas MY09/MY11 is less sensitive for HPV 31 [29].

Nearly a third of HPV-positive samples in our series were detected in patients with normal cytology. This prevalence is higher than that of other Spanish studies. This might be due to the fact that the women in our study had an abnormal Pap test and attended a specialised gynaecology unit for cervical pathology.

In NC samples, HPV 16 was the most common genotype (21%), followed by HPV 53 (16%). The HPV distribution found in NC cases is similar to that reported by Conesa et al. [26] from a cohort of women from Eastern Spain. The same distribution was found in ASCUS and LSIL samples with HPV 16 (31% and 26%, respectively) being the most prevalent, followed by HPV 53 (16% and 19%, respectively). The high prevalence of HPV 53 in ASCUS and LSILs is similar to that in other studies from Spain and France.

In HSILs, the most frequent type was HPV 16 (51%), followed by 52 (14%) and 31 (11%). In our series, HPV 53 was not detected in HSILs, suggesting that it is not a high-risk type. An interesting finding of our study was that HPV 52 was the second most common genotype in HSIL cases, whereas its prevalence in NC and LSILs was lower.

Recently, multiple HPV infections have been analysed because, with the development of anti-HPV vaccines not covering all genotypes, the distribution of infection with types not covered by vaccines could be affected. The elimination of one HPV type could affect the natural history of the remaining genotypes. Therefore, obtaining a solid knowledge of genotype HPV distribution is becoming imperative. Multiple types of HPV have been described in up to 50% of NC and LSIL cases in the population in Northwest Spain [15], in accordance with other studies [30].

In the present study, multiple infections were detected in 34% of samples, and were more frequent in younger women (< 33 years). This observation is consistent with the results of Mejlhede et al. [8], and supports the fact that greater sexual activity in younger women may be associated with the transmission of multiple HPV types. The presence of co-infection was more frequent in LSIL cases (45%), followed ASCUS (36%) and NC (25%), whereas a decreased frequency of co-infection was found in HSILs (20%). Similar to other Spanish studies [15,26], in our series, ASCUS/LSILs were associated with co-infections, whereas HSILs were associated with single infection (p < 0.01). It is still not clear whether co-infection with several types increases the risk of progression. It seems that, as lesions progress from low to high grade, high-risk oncogenic types may persist, whereas less oncogenic types are eliminated [26].

## Conclusions

In conclusion, this study demonstrates an important prevalence of high- and low-risk HPV genotypes even in patients with no evidence of cytological anomalies, as reported by Krambeck et al. [31]. We also demonstrated a high incidence of HPV co-infection, mainly in LSILs. It is important to detect the specific genotype for two reasons: (1) to differentiate persistent and new infections; and (2) to increase our knowledge of the epidemiological distribution because it is a necessary basis to predict the effect of vaccines on the incidence of infection, and to evaluate the cross-protection from current vaccines.

## Competing interests

The authors declare that they have no competing interests.

## Authors' contributions

PM designed the experiments, performed analysis and interpretation of data, and drafted the manuscript; LK and DG performed genotyping methods; AMLG performed pathological diagnosis; MJMA performed data analysis; and CB performed pathological diagnosis and supervised the study. VA performed statistical treatment of data.

All authors read and approved the final manuscript.

## Pre-publication history

The pre-publication history for this paper can be accessed here:

http://www.biomedcentral.com/1471-2334/11/316/prepub
